# Development of microRNA therapeutics is coming of age

**DOI:** 10.15252/emmm.201100899

**Published:** 2014-06-16

**Authors:** Eva van Rooij, Sakari Kauppinen

**Affiliations:** 1 Hubrecht Institute, KNAW and University Medical CenterUtrecht, The Netherlands; 2 Department of Clinical Medicine, Aalborg UniversityAalborg, Denmark; 3 Department of Haematology, Aalborg University HospitalAalborg, Denmark

**Keywords:** antimiR, mimic, miRNA, therapeutics

## Abstract

MicroRNAs (miRNAs) play key regulatory roles in diverse biological processes and are frequently dysregulated in human diseases. Thus, miRNAs have emerged as a class of promising targets for therapeutic intervention. Here, we describe the current strategies for therapeutic modulation of miRNAs and provide an update on the development of miRNA-based therapeutics for the treatment of cancer, cardiovascular disease and hepatitis C virus (HCV) infection.

See also Glossary for abbreviations used in this article

## Introduction

**Figure 1:**
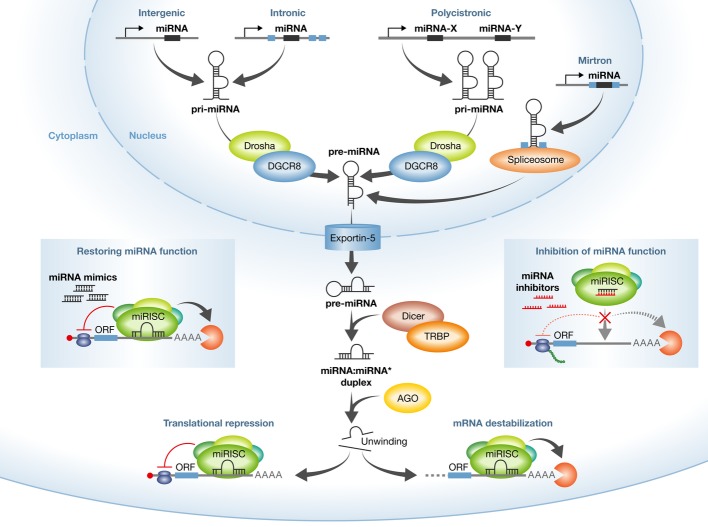
miRNA biogenesis and modulation of miRNA activity by miRNA mimics and antimiR oligonucleotides MiRNA genes are transcribed by RNA polymerase II from intergenic, intronic or polycistronic loci to long primary miRNA transcripts (pri-miRNAs) and processed in the nucleus by the Drosha–DGCR8 complex to approximately 70 nt pre-miRNA hairpin structures. The most common alternative miRNA biogenesis pathway involves short intronic hairpins, termed mirtrons, that are spliced and debranched to form pre-miRNA hairpins. Pre-miRNAs are exported into the cytoplasm and then cleaved by the Dicer–TRBP complex to imperfect miRNA: miRNA* duplexes about 22 nucleotides in length. In the cytoplasm, miRNA duplexes are incorporated into Argonaute-containing miRNA-induced silencing complex (miRISC), followed by unwinding of the duplex and retention of the mature miRNA strand in miRISC, while the complementary strand is released and degraded. The mature miRNA functions as a guide molecule for miRISC by directing it to partially complementary sites in the target mRNAs, resulting in translational repression and/or mRNA degradation. Currently, two strategies are employed to modulate miRNA activity: restoring the function of a miRNA using double-stranded miRNA mimics, and inhibition of miRNA function using single-stranded antimiR oligonucleotides.

MicroRNAs (miRNAs) are a class of short (∼22 nt) endogenous non-coding RNAs that mediate post-transcriptional regulation of gene expression (Ambros, 2004; Bartel, 2009). Since the discovery of the first miRNAs, lin-4 and let-7, in the nematode *Caenorhabditis elegans* (Lee *et al*, 1993; Wightman *et al*, 1993; Reinhart *et al*, 2000), 30424 mature miRNAs have been annotated in 206 species according to the miRBase Sequence Database release 20 of June 2013 (Kozomara & Griffiths-Jones, 2011). Most miRNAs are transcribed by RNA polymerase II from intergenic, intronic or polycistronic loci to long primary transcripts, called pri-miRNAs (Fig 1). Pri-miRNAs are processed sequentially first in the nucleus by the Drosha–DGCR8 complex to approximately 70 nt pre-miRNA hairpin structures and then in the cytoplasm by the Dicer–TRBP complex to approximately 22 nt miRNA duplexes (Fig 1) (Bernstein *et al*, 2001; Grishok *et al*, 2001; Hutvágner *et al*, 2001; Lee *et al*, 2003, 2004; Yi *et al*, 2003; Bohnsack *et al*, 2004; Denli *et al*, 2004). In addition to the canonical miRNA biogenesis pathway, many Drosha–DGCR8-independent pathways can produce pre-miRNAs. The most common alternative pathway involves short intronic hairpins, termed mirtrons, that are spliced and debranched to form pre-miRNA hairpins (Fig 1) (Yang & Lai, 2011; Ladewig *et al*, 2014). In the cytoplasm, miRNA duplexes are incorporated into an Argonaute protein-containing miRNA-induced silencing complex (miRISC), followed by unwinding of the duplex and retention of the mature miRNA strand in miRISC, while the complementary strand is released and degraded (Fig 1) (Carthew & Sontheimer, 2009; Krol *et al*, 2010).

Metazoan miRNAs guide the miRISC to target mRNAs by base pairing imperfectly with their 3′ untranslated regions (UTRs), leading to translational repression and/or degradation of the mRNA targets (Fig 1) (Krol *et al*, 2010; Huntzinger & Izaurralde, 2011). This interaction is nucleated by perfect base pairing of the miRNA seed region (nucleotides 2-7 in the mature miRNA) with a complementary seed match site in the 3′ UTR of the target mRNA (Bartel, 2009). Apart from canonical seed match sites, other types of miRNA-binding sites, such as centered sites, 3′ supplementary sites and bulged sites have also been described (Bartel, 2009; Shin *et al*, 2010; Chi *et al*, 2012). Computational prediction of target mRNAs with conserved seed sites, combined with genome-wide surveys for additional types of binding sites, suggests that >60% of all mammalian protein-coding genes can be regulated by miRNAs (Bartel, 2009; Friedman *et al*, 2009). Indeed, miRNAs have been implicated in the regulation of many cellular and developmental processes (Kloosterman & Plasterk, 2006; Bushati & Cohen, 2007; Braun & Gautel, 2011). Furthermore, miRNA deregulation is a common feature in cancer, CNS disorders, inflammation, cardiovascular diseases and metabolic disorders, suggesting that miRNAs could serve as targets for therapeutic intervention (van Rooij *et al*, 2007; Gottwein & Cullen, 2008; Ventura & Jacks, 2009; Najafi-Shoushtari *et al*, 2010; Rayner *et al*, 2010; Grueter *et al*, 2012; van Rooij, 2012; van Rooij & Olson, 2012; Rottiers & Näär, 2012; Salta & De Strooper, 2012; Stenvang *et al*, 2012; Thorsen *et al*, 2012). Hence, there are currently many efforts focusing on the development of miRNA therapeutics for the treatment of a wide array of human diseases.

This review will focus on recent progress in the field of miRNA therapeutics. We will describe the current strategies for therapeutic modulation of miRNA activity *in vivo*. Furthermore, we will discuss the use of miRNAs as a therapeutic modality and provide an update on the development of miRNA-based therapies for treatment of cancer, cardiovascular disease and HCV infection.

## Therapeutic modulation of miRNA activity

An expanding inventory of genetic gain- or loss-of-function studies of specific miRNAs together with recent data from pharmacological modulation of individual miRNAs or miRNA families in animal disease models implies that miRNAs are viable targets for therapeutics (van Rooij, 2012; van Rooij & Olson, 2012; Stenvang *et al*, 2012; Thorsen *et al*, 2012). Indeed, miRNAs have many advantages as a therapeutic modality. The mature miRNA sequences are short and often completely conserved across multiple vertebrate species. These characteristics make miRNAs relatively easy to target therapeutically and allows for using the same miRNA-modulating compound in preclinical efficacy and safety studies as well as in clinical trials. Moreover, miRNAs have typically many targets within cellular networks, which, in turn, enable modulation of entire pathways in a disease state via therapeutic targeting of disease-associated miRNAs. Currently, two approaches are employed to modulate miRNA activity: (i) restoring the function of a miRNA using either synthetic double-stranded miRNAs or viral vector-based overexpression and (ii) inhibiting the function of a miRNA using chemically modified antimiR oligonucleotides (Fig 1). The next section will describe the design of synthetic miRNA mimics and chemically modified antimiRs and their use for modulating disease-implicated miRNAs.

## Restoring miRNA function

**Figure 2:**
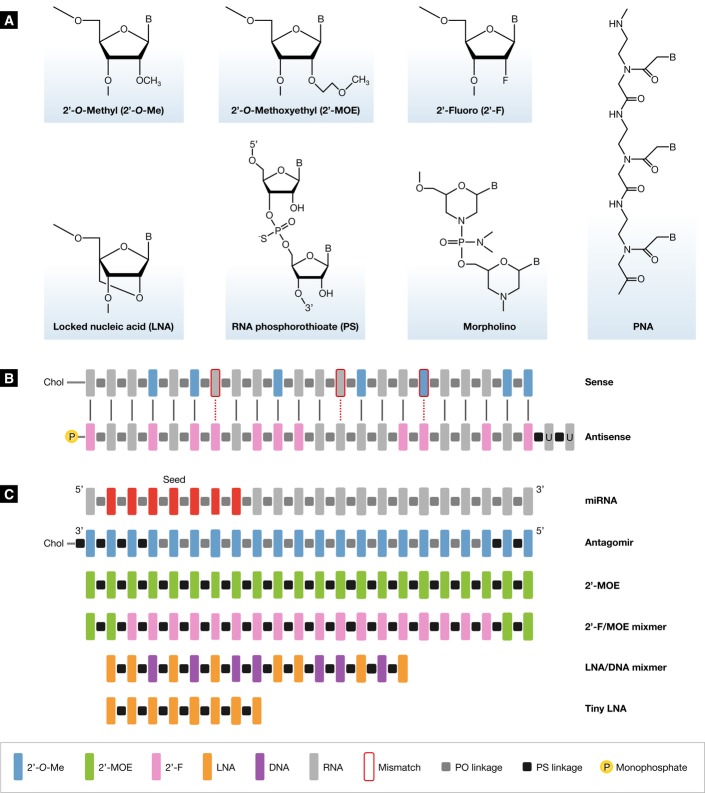
Design of chemically modified miRNA modulators (A) Structures of chemical modifications used in miRNA modulators. A number of different sugar modifications are used to increase the duplex melting temperature (*T*_m_) of antimiR oligonucleotides. The 2′-O-methyl (2′-O-Me), 2′-O-methoxyethyl (2′-MOE) and 2′-fluoro (2′-F) nucleotides are modified at the 2′ position of the sugar moiety, whereas locked nucleic acid (LNA) is a bicyclic RNA analogue in which the ribose is locked in a C3′-endo conformation by introduction of a 2′-O,4′-C methylene bridge. To increase nuclease resistance and enhance the pharmacokinetic properties, most antimiR oligonucleotides harbor phosphorothioate (PS) backbone linkages, in which sulfur replaces one of the non-bridging oxygen atoms in the phosphate group. In morpholino oligomers, a six-membered morpholine ring replaces the sugar moiety. Morpholinos are uncharged and exhibit a slight increase in binding affinity to their cognate miRNAs. PNA oligomers are uncharged oligonucleotide analogues, in which the sugar–phosphate backbone has been replaced by a peptide-like backbone consisting of N-(2-aminoethyl)-glycine units. (B) An example of a synthetic double-stranded miRNA mimic described in this review. One way to therapeutically mimic a miRNA is by using synthetic RNA duplexes that harbor chemical modifications for improved stability and cellular uptake. In such constructs, the antisense (guide) strand is identical to the miRNA of interest, while the sense (passenger) strand is modified and can be linked to a molecule, such as cholesterol, for enhanced cellular uptake. The sense strand contains chemical modifications to prevent miRISC loading. Several mismatches can be introduced to prevent this strand from functioning as an antimiR, while it is further left unmodified to ensure rapid degradation. The 2′-F modification helps to protect the antisense strand against exonucleases, hence making the guide strand more stable, while it does not interfere with miRISC loading. (C) Design of chemically modified antimiR oligonucleotides described in this review. Antagomirs are 3′ cholesterol-conjugated, 2′-O-Me oligonucleotides fully complementary to the mature miRNA sequence with several PS moieties to increase their *in vivo* stability. The use of unconjugated 2′-F/MOE-, 2′-MOE- or LNA-modified antimiR oligonucleotides harboring a complete PS backbone represents another approach for inhibition of miRNA function *in vivo*. The high duplex melting temperature of LNA-modified oligonucleotides allows efficient miRNA inhibition using truncated, high-affinity 15–16-nucleotide LNA/DNA antimiR oligonucleotides targeting the 5′ region of the mature miRNA. Furthermore, the high binding affinity of fully LNA-modified 8-mer PS oligonucleotides, designated as tiny LNAs, facilitates simultaneous inhibition of entire miRNA seed families by targeting the shared seed sequence.

One strategy to therapeutically restore the activity of a miRNA is to use synthetic RNA duplexes that harbor chemical modifications to improve stability and cellular uptake (Garzon *et al*, 2010; Bader *et al*, 2011; Thorsen *et al*, 2012) (Fig 2A). In such double-stranded miRNA mimics, the strand identical to the miRNA of interest is the guide (antisense) strand, while the opposite (passenger or sense) strand is less stable and can be linked to a molecule, such as cholesterol, to enhance cellular uptake (Fig 2B). In addition, the passenger strand may contain chemical modifications to prevent RISC loading, while it is further left unmodified to ensure rapid degradation (Chen *et al*, 2008). Since the miRISC needs to recognize the guide strand as a miRNA, the chemical modifications that can be used are limited. The 2′-fluoro (2′-F) modification helps to protect against exonucleases, hence making the guide strand more stable, while it does not interfere with RISC loading (Chiu & Rana, 2003). An example of a potential mimic design is shown in Fig 2B. It should be noted that double-stranded miRNA mimics can potentially induce a non-specific interferon response through Toll-like receptors (Peacock *et al*, 2011). Another potential issue with miRNA replacement therapies is the challenge of restoring the level of a down-regulated miRNA, while preventing the introduction of supraphysiological levels of the miRNA. Additionally, while miRNA mimicry increases the levels of a miRNA that is lost during disease progression, systemic delivery of such miRNA mimics can also result in uptake by non-target tissues that normally do not express the miRNA of interest, resulting in potential off-target effects. Thus, targeted delivery of miRNA mimics to the appropriate cell or tissue type is going to be important to prevent unwanted side effects of this therapeutic approach. In addition to chemically modified miRNA mimics, the use of lenti-, adeno- or adeno-associated viruses (AAV) to drive expression of a given miRNA for restoring its activity has been successfully reported by several studies (Kota *et al*, 2009; Trang *et al*, 2010; Miyazaki *et al*, 2012). This strategy is discussed in more detail below.

## Inhibition of miRNA function

Mature miRNAs can be inhibited using either miRNA sponges or antisense oligonucleotides, known as antimiRs. A miRNA sponge uses transgenic overexpression of RNA molecules harboring complementary binding sites to a miRNA of interest to block the function of a given miRNA or a miRNA family (Ebert & Sharp, 2010). While this approach has shown great utility as an experimental tool, antimiRs have shown greater promise from a therapeutic perspective.

Efficient silencing of dysregulated miRNAs *in vivo* requires that the antimiR oligonucleotides are chemically modified to improve their binding affinity, biostability and pharmacokinetic properties. Additionally, since miRNA expression levels vary greatly depending on the cell and tissue type as well as disease, extensive preclinical studies in animal disease models are needed to determine the optimal level of inhibition for a given miRNA target. The most commonly used sugar modifications for increasing the duplex melting temperature (*T*_m_) and improving nuclease resistance of antimiRs include the 2′-*O*-methyl (2′-*O*-Me), 2′-*O*-Methoxyethyl (2′-MOE) 2′-fluoro and the bicyclic locked nucleic acid (LNA) modifications, respectively, (Fig 2A) (Davis *et al*, 2006, 2009; Elmén *et al*, 2008b; Esau, 2008; Stenvang & Kauppinen, 2008; Lennox & Behlke, 2010, 2011; van Rooij & Olson, 2012; Stenvang *et al*, 2012). Among the different sugar modifications, LNA exhibits the highest affinity toward complementary RNA with an increase in *T*_m_ of +2–8°C per introduced LNA modification (Braasch & Corey, 2001; Petersen & Wengel, 2003).

Increased nuclease resistance is achieved by substituting the phosphodiester (PO) backbone linkages with phosphorothioate (PS) linkages in antimiR oligonucleotides (Lennox & Behlke, 2010), or by using peptide nucleic acid (PNA) or morpholino oligomers, respectively, designed to target miRNAs (Flynt *et al*, 2007; Kloosterman *et al*, 2007; Martello *et al*, 2007; Fabani & Gait, 2008; Fabani *et al*, 2010; Babar *et al*, 2012; Torres *et al*, 2012) (Fig 2A). Apart from nuclease resistance, PS backbone modifications also enhance binding to plasma proteins, leading to reduced clearance by glomerular filtration and urinary excretion. Thus, PS-modified oligonucleotides exhibit markedly improved pharmacokinetic properties, facilitating their delivery into many peripheral tissues *in vivo* (Levin, 1999). PNA oligomers are uncharged oligonucleotide analogues, in which the sugar–phosphate backbone has been replaced by a peptide-like backbone consisting of N-(2-aminoethyl)-glycine units (Egholm *et al*, 1992) (Fig 2A). Polylysine-conjugated and nanoparticle-encapsulated PNA antimiRs have been shown to efficiently inhibit miRNA function in cultured cells and in mice (Fabani & Gait, 2008; Fabani *et al*, 2010; Babar *et al*, 2012; Torres *et al*, 2012). Morpholinos are uncharged and with a slightly increased binding affinity to complementary miRNAs (Flynt *et al*, 2007; Kloosterman *et al*, 2007; Martello *et al*, 2007) (Fig 2A).

The first approach to inhibit miRNA function *in vivo* was to use cholesterol-conjugated antagomirs or chemically modified antimiR oligonucleotides that were fully complementary to the mature miRNA sequence (Krützfeldt *et al*, 2005, 2007; Esau *et al*, 2006) (Fig 2C). Indeed, two studies showed that truncation of a cholesterol-conjugated 2′-*O*-Me-modified antagomir-122 or a uniform 2′-MOE-modified antimiR-21, by 3 or more nucleotides results in loss of *in vitro* and *in vivo* efficacy (Davis *et al*, 2006; Krützfeldt *et al*, 2007). However, more recent studies have shown that the LNA chemistry enables design of truncated LNA-modified antimiRs of 15–16 nucleotides in length with high affinity toward their cognate miRNA targets and high potency in cell culture and *in vivo* (Elmén *et al*, 2008a,b; Worm *et al*, 2009; Najafi-Shoushtari *et al*, 2010; Boon *et al*, 2011; Eskildsen *et al*, 2011; Montgomery *et al*, 2011; Porrello *et al*, 2011; Caruso *et al*, 2012; Grueter *et al*, 2012). In addition, Obad *et al* (2011) developed an approach for inhibiting miRNA seed families using ultra-short LNA oligonucleotides that base pair with the seed region, based on the high duplex melting temperature of fully LNA-modified 8-mer PS oligonucleotides (Fig 2C). Several studies have now shown that pharmacological inhibition of miRNA function using 8-mer LNAs can result in a therapeutic benefit in mouse disease models *in vivo* (Garchow *et al*, 2011; Hullinger *et al*, 2012; Leucci *et al*, 2012; Ranganathan *et al*, 2012; Zhang *et al*, 2012). A potential advantage of this approach is that unlike longer antimiRs, it enables antagonism of disease-implicated miRNA family members that may have overlapping roles in disease, which was recently addressed in two studies in mice. Hullinger *et al* showed that an 8-mer LNA complementary to the seed region of the miR-15 family members, including miR-15a, -15b, 16-1, -16-2, -195 and miR-497, was more potent in eliciting derepression of downstream targets than a 16-mer LNA-modified antimiR targeting a specific family member, while both antimiR compounds showed comparable uptake to cardiac tissue (Hullinger *et al*, 2012). Notably, pharmacological inhibition of the miR-15 family by the 8-mer antimiR reduced infarct size and cardiac remodeling and led to enhanced cardiac function in response to myocardial infarction (MI). The second study asked whether inhibition of the miR-34 family (miR-34a, -34b and -34c) by a subcutaneously delivered 8-mer LNA could provide a therapeutic benefit in mice with preexisting pathological cardiac remodeling and dysfunction due to MI (Bernardo *et al*, 2012). Indeed, the seed-targeting 8-mer LNA was effective in inhibiting all three miR-34 family members in two different cardiac stress models and attenuated cardiac remodeling and atrial enlargement, whereas inhibition of miR-34a alone with a 15-mer LNA-modified antimiR provided no benefit in the MI model (Bernardo *et al*, 2012).

## Delivery of miRNA modulators

The main challenge for development of miRNA-based therapeutics is efficient and safe delivery of miRNA mimics and antimiRs. Two strategies have been used to deliver miRNA replacement therapies *in vivo*: (i) formulated, synthetic, double-stranded miRNA mimics, and (ii) viral constructs over-expressing the lost or down-regulated miRNA. Intravenously and intratumorally injected miRNA mimics complexed with liposome nanoparticles (Pramanik *et al*, 2011), polyethyleneimine (Ibrahim *et al*, 2011) or atelocollagen (Takeshita *et al*, 2010) have been used to restore the functions of various tumor-suppressive miRNAs in mouse cancer models. Notably, the first liposome-formulated mimic is currently being tested in a Phase I Clinical Trial in patients with unresectable primary liver cancer (http://www.mirnatherapeutics.com; http://www.clinicaltrials.gov). Furthermore, intranasally administered lentivirus-based expression constructs were utilized to deliver tumor-suppressive miRNAs *in vivo* (Trang *et al*, 2010), whereas two reports demonstrated the efficacy of systemically delivered adeno-associated virus (AAV) vector-based miRNA expression constructs. In the first study, Kota *et al* reported on AAV-mediated delivery of miR-26a, which blunted tumorigenesis in a mouse model of hepatocellular carcinoma (Kota *et al*, 2009), while Miyazaki *et al* showed that AAV-based delivery of miR-196a inhibits the decay of the androgen receptor, thereby reducing spinal and bulbar muscular atrophy (Miyazaki *et al*, 2012). Currently, there are several AAV serotypes available that can be used for tissue enrichment based on natural tropism toward specific cell types and interaction between different cellular receptors and serotypes. Additionally, tissue-specific promoters allow for tissue-specific expression of the miRNA, which can further enhance tissue- or cell-specific delivery. AAV-based constructs are currently being used in several clinical trials for gene therapy, and the safety profiles are thus far encouraging (Aalbers *et al*, 2011).

Two strategies have been utilized to enhance *in vivo* delivery of antimiR oligonucleotides; (i) cholesterol conjugation and (ii) modification of the phosphate backbone with PS linkages. The 3′ cholesterol-conjugated, 2′-*O*-Me-modified antagomirs (Fig 2C) have become a well-validated experimental tool for *in vivo* inhibition of miRNAs, since this approach was first described in mice by Krützfeldt *et al* (2005). In this report, intravenously (i.v.) injected antagomirs showed a broad biodistribution and miRNA silencing in many mouse tissues *in vivo*. In addition, effective inhibition of the liver-expressed miR-122 was achieved in mice by three intravenous (i.v.) doses of 80 mg/kg antagomir-122, which resulted in derepression of direct miR-122 targets in the liver and lowering of serum cholesterol by 40% (Krützfeldt *et al*, 2005). Additional studies showed that antagomirs localize in a cytosolic compartment, distinct from P-bodies, and implied that antagomirs promote degradation of the targeted miRNA (Krützfeldt *et al*, 2007). In addition to efficacy in the liver, antagomirs have now shown pharmacological activity in many other tissues as well (reviewed in: Thum, 2012).

Additionally, PS backbone linkages can be employed to enhance the pharmacokinetic properties of antisense oligonucleotides (Levin, 1999). The antagomir approach contains 2 PS modifications at the 5′ end and 4 at the 3′ end, which have been shown to be important for their *in vivo* activity, whereas complete replacement of the phosphodiester (PO) backbone by PS linkages decreased antagomir efficiency (Krützfeldt *et al*, 2007) (Fig 2C). By contrast, efficient inhibition of miRNA function has been achieved using saline-formulated, chemically modified antimiRs harboring a complete PS backbone (Fig 2C). An increasing number of reports have described silencing of miRNAs *in vivo* by unconjugated LNA-modified antimiRs ranging from 8 nt to 16 nt in length as described in the previous section. Administration of such antimiRs in mice either by i.v., intraperitoneal or subcutaneous injections resulted in antimiR uptake in the tissue of interest, which led to inhibition of miRNA function and derepression of direct target mRNAs. In addition to high accumulation in the kidney and liver, antimiR uptake and pharmacological activity have also been reported in other peripheral tissues, such as heart, lung, spleen and bone marrow (Elmén *et al*, 2008a,b; Worm *et al*, 2009; Obad *et al*, 2011; Hildebrandt-Eriksen *et al*, 2012; Hullinger *et al*, 2012; Ranganathan *et al*, 2012; Zhang *et al*, 2012). However, the mechanisms of cellular uptake and distribution are still poorly understood.

The utility of device-based approaches to establish effective local delivery of miRNA therapeutics was recently assessed in the study by Hinkel *et al* (2013), in which the efficacy of antimiR-92 was compared after systemic and catheter-based delivery, respectively, in a porcine model of ischemia and reperfusion. Interestingly, catheter-based delivery of antimiR-92a significantly reduced infarct size and improved cardiac function, whereas systemic delivery of antimiR-92a did not (Hinkel *et al*, 2013), thereby demonstrating a benefit of local delivery of antimiR-92a in the setting of cardiac ischemic injury.

## miRNA-based therapeutics: from bench to bedside

Recent work has shown that miRNAs are frequently deregulated in human diseases, suggesting that they could serve as viable targets for development of miRNA-based therapeutics (Mendell & Olson, 2012; van Rooij, 2012; van Rooij & Olson, 2012; Stenvang *et al*, 2012; Thorsen *et al*, 2012) (Table 1). In this section, we review selected examples, in which pharmacological modulation of miRNA activity has demonstrated a therapeutic benefit for the treatment of cancer, heart failure, atherosclerosis and HCV infection, respectively.

**Table 1:** MicroRNA-based therapeutics in development

Company	miRNA target	Mode of action	Indication	Status
Santaris Pharma	miR-122	antimiR	HCV	Clinical Phase II

Mirna Therapeutics	miR-34	mimic	Unresectable primary liver cancer	Clinical Phase I
	
	let-7	mimic	Cancer	Preclinical

Regulus Therapeutics	miR-122	antimiR	HCV	Clinical Phase I
	
	miR-221	antimiR	Hepatocellular carcinoma	Preclinical
	
	miR-10b	antimiR	Glioblastoma	Preclinical
	
	miR-21	antimiR	Hepatocellular carcinoma	Preclinical
	
	miR-21	antimiR	Kidney fibrosis	Preclinical
	
	miR-33	antimiR	Atherosclerosis	Preclinical

miRagen Therapeutics	miR-208	antimiR	Heart failure	Preclinical
	
	miR-15/195	antimiR	Post-MI remodeling	Preclinical
	
	miR-145	antimiR	Vascular disease	Preclinical
	
	miR-451	antimiR	Myeloproliferative disease	Preclinical
	
	miR-29	mimic	Fibrosis	Preclinical
	
	miR-208	antimiR	Cardiometabolic disease	Preclinical
	
	miR-92	antimiR	Peripheral artery disease	Preclinical

## miR-34-based cancer therapeutics

Most studies on modulation of miRNA activity for cancer therapeutics have focused on miRNA replacement therapies to reintroduce miRNAs that are either lost or down-regulated in cancer cells. The miR-34 family of miRNAs is consistently down-regulated in a broad range of malignancies. This family comprises three members, miR-34a, -34b and 34c, and based on the shared seed sequence, they are predicted to control an overlapping set of target mRNAs and are, thus, likely to be functionally redundant (He *et al*, 2007). The miR-34 family has been shown to control cellular proliferation, cell cycle and apoptosis. Notably, p53, a well-known tumor suppressor that plays a key role in suppressing cancer by regulating cell cycle, apoptosis and DNA repair, has been shown to transcriptionally activate the expression of all miR-34 family members (Chang *et al*, 2007). On the other hand, miR-34 can stimulate p53 activity by targeting and down-regulating SIRT1, an NAD^+^-dependent lysine deacetylase that removes protective acetyl groups on p53, causing p53 ubiquitylation and proteasome-mediated degradation (Yamakuchi *et al*, 2008), thereby establishing a positive feedback loop. The strong tumor-suppressive effects observed for miR-34 are likely due to the combined modulation of several target mRNAs involved in different oncogenic processes, rather than regulation of a single target, since none of the miR-34 target mRNAs alone can fully recapitulate the miR-34 loss-of-function phenotype (Kaller *et al*, 2011).

Based on its strong tumor-suppressive effects *in vitro*, many efforts have focused on increasing miR-34 levels in cancer cells by using miRNA mimics. In most cases, the miR-34 mimic was delivered directly by intratumoral injections, which is only therapeutically feasible for easily accessible and localized tumors that have not yet metastasized (Wiggins *et al*, 2010). Since unformulated miRNA mimics are rapidly degraded *in vivo*, an optimized, lipid-based formulation can be used to enhance delivery. Liposomal encapsulation of miRNA mimics has been shown to facilitate cellular uptake by endocytosis and protect the constructs from degradation (Wiggins *et al*, 2010). However, positively charged lipids have been shown to induce dose-dependent toxicities and an interferon response (Pecot *et al*, 2011). To circumvent these side effects, the utility of neutral lipid emulsion (NLE) has been explored. NLE is anionic at normal body pH (7-7.5), which potentially prevents unwanted interactions with the negative charge of cellular membranes in the endothelium or other tissues. However, in a tumor area, where the pH tends to be lower, the lipids become cationic, which enhances uptake into tumor cells (Wiggins *et al*, 2010). Indeed, Trang *et al* (2011) showed that systemically delivered synthetic miRNA mimics complexed with NLE are preferentially targeted to lung tumors and show a therapeutic benefit in mouse models of lung cancer. Therapeutic delivery was demonstrated using mimics of the tumor-suppressor miRNAs, miR-34a and let-7, both of which are often down-regulated or lost in lung cancer. Systemic treatment with a formulated miR-34 mimic in an orthotopic Kras-activated mouse model of non-small cell lung cancer (NSCLC) led to a significant decrease in tumor burden with a 60% reduction in tumor area compared to mice treated with a control construct. Similar results were obtained with NLE-complexed let-7 mimics (Trang *et al*, 2011). Moreover, administration of such lipid-based miRNA mimic formulations did not elicit an immune response and showed no increase in kidney or liver enzymes, suggesting that this strategy is well tolerated *in vivo* (Trang *et al*, 2011). In May 2013, Mirna Therapeutics announced the commencement of a phase 1 study of the liposome-formulated miR-34 mimic-based drug, designated as MRX34, in patients with primary liver cancer or metastatic cancer with liver involvement. This is the first miRNA mimic to advance into the clinic and, thus, an important milestone for the development of miRNA-based replacement therapeutics (Bouchie, 2013).

## Targeting of miR-33 for the treatment of atherosclerosis

A number of recent reports have shown that the human sterol-regulatory-element-binding-protein genes SREBF1 and SREBF2 harbor two intronic miRNAs, miR-33b and miR-33a, respectively, which regulate cholesterol, fatty acid and triglyceride homeostasis in concert with their host gene products, SREBP1 and SREBP2 (Gerin *et al*, 2010; Horie *et al*, 2010; Marquart *et al*, 2010; Najafi-Shoushtari *et al*, 2010; Rayner *et al*, 2010, 2011a). The miR-33a and miR-33b sequences share the same seed region and are thus predicted to regulate an overlapping set of target mRNAs, indicating that they may have redundant biological functions. Interestingly, mice and other rodents have only one miR-33 isoform in intron 16 of SREBF2, corresponding to miR-33a in humans and non-human primates (Rottiers & Näär, 2012).

The miR-33a/b family plays an important role in post-transcriptional repression of the ATP-binding cassette transporter ABCA1, which is essential for high-density lipoprotein (HDL) biogenesis and promotes reverse cholesterol transport from peripheral tissues, such as atherogenic macrophages, back to the liver (Rottiers & Näär, 2012). Several studies have shown that genetic deletion or antimiR-mediated inhibition of miR-33 in mice leads to derepression of hepatic ABCA1 and increase in circulating HDL cholesterol levels by up to 40%, suggesting that silencing of miR-33 could be a useful therapeutic strategy for atherosclerosis (Horie *et al*, 2010; Marquart *et al*, 2010; Najafi-Shoushtari *et al*, 2010; Rayner *et al*, 2010, 2011a). Notably, inhibition of miR-33 by a subcutaneously delivered 2′F/MOE-modified antimiR for 4 weeks in hyperlipidemic low-density lipoprotein receptor (*Ldlr*^−/−^) knockout mice fed a standard chow diet enhanced reverse cholesterol transport and showed atherosclerotic plaque regression, consistent with accumulation of the antimiR-33 in plaque macrophages (Rayner *et al*, 2011a). These findings were corroborated by two subsequent reports. Horie *et al* (2012) showed that genetic loss of miR-33 in apolipoprotein E-deficient (*Apoe*^−/−^) knockout mice enhanced cholesterol efflux and significantly reduced atherosclerotic plaque size and lipid content, whereas Rotllan *et al* (2013) reported that long-term inhibition of miR-33 in *Ldlr*^−/−^ knockout mice fed a Western diet significantly reduced the progression of atherosclerosis. By comparison, a third study by Marquart *et al* (2013) reported that long-term inhibition of miR-33 in high-fat-/high-cholesterol-fed *Ldlr*^−/−^ mice failed to sustain elevated HDL cholesterol levels in the serum and did not alter progression of atherosclerosis, despite initial increase in HDL cholesterol after 2 weeks of treatment (Marquart *et al*, 2013). This observed discrepancy could, at least in part, be due to the high excess of dietary cholesterol (1.25%) used in the study by Marquart *et al* (2013). Nevertheless, the different outcomes described above raise concerns with regard to translating pharmacology data from miR-33 inhibition studies in mouse models to human therapy and highlight the need of additional long-term studies in larger animals, which in contrast to mice, harbor both miR-33 isoforms. Indeed, two studies have reported on pharmacological inhibition of miR-33 in non-human primates. Rayner *et al* (2011b) showed that treatment of normal male African green monkeys by a subcutaneously delivered 2′F/MOE-modified antimiR targeting both miR-33a and miR-33b resulted in derepression of hepatic ABCA1 levels and sustained increase in plasma HDL cholesterol over 12 weeks (Rayner *et al*, 2011b). In addition, pharmacological inhibition of miR-33a/b led to derepression of several miR-33 targets implicated in fatty acid oxidation and a decrease in very-low-density lipoprotein (VLDL) triglycerides, without any evidence for adverse effects in the treated monkeys (Rayner *et al*, 2011b). Recently, Rottiers *et al* (2013) reported on pharmacological inhibition of the miR-33 family using a subcutaneously injected, seed-targeting 8-mer LNA-modified antimiR in a non-human primate metabolic disease model. In this study, treatment of obese and insulin-resistant female African green monkeys with the 8-mer antimiR over 108 days resulted in derepression of direct miR-33 targets, including ABCA1, increased circulating HDL cholesterol and was well tolerated without any adverse effects. These findings demonstrate for the first time the utility of seed-targeting antimiRs in pharmacological inhibition of an entire miRNA family in non-human primates and imply that even under conditions of obesity, hyperglycemia and low insulin responsiveness in a severe metabolic disease animal model, inhibition of the miR-33 family is a feasible approach to increase circulating HDL cholesterol (Rottiers *et al*, 2013).

## Inhibition of miR-208 for the treatment of heart failure and diabetes

The α-myosin heavy chain (αMHC) gene is one of the most important genes for determining cardiomyocyte contractility. Several years ago, it was discovered that this gene not only gives rise to a key protein, but additionally produces a miRNA, known as miR-208a (van Rooij *et al*, 2007). Although the expression level of miR-208a does not change significantly during cardiac stress, this miRNA appears to play a key role in the stress-induced induction of βMHC, the pathological counterpart of αMHC and is, thus, a relevant player during pathological remodeling that occurs during cardiac disease (van Rooij *et al*, 2007). Efficacy studies in animal models of heart disease using an LNA-modified antimiR targeting miR-208a indicated that subcutaneous delivery of antimiR-208a prevents disease-related cardiac remodeling, a decline in function, and death during diastolic heart disease (Montgomery *et al*, 2011). These studies underscore the potential of antimiR-based therapies for modulating cardiac miRNAs and validate miR-208 as a therapeutic target for the modulation of cardiac function and remodeling during heart disease.

Follow-up studies showed that long-term treatment with antimiR-208 prevented age-induced weight gain normally observed in mice. This effect occurred in the absence of detectable toxicity or observable cardiac effects. To further investigate this phenotype, the effect of antimiR-208a in a model of type II diabetes (high-fat (HF) diet) was tested, which indicated that mice on HF diet and treated with antimiR-208a showed a remarkable dose-dependent reduction in the increase in body weight. This effect was due to a reduction in fat weight in animals treated with antimiR-208 compared to animals treated with either saline or a control oligonucleotide. Additionally, while HF diet-induced obesity resulted in glucose intolerance in untreated mice, antimiR-208a treated mice showed a normalized glucose response, as measured by a glucose tolerance test (Grueter *et al*, 2012). These findings imply that inhibition of miR-208, in addition to blocking cardiac remodeling in the setting of heart disease, can have profound effects on metabolism and imply that the heart plays an unexpected role in the regulation of systemic metabolism and energy expenditure based on a miR-208-dependent mechanism.

Shortly after the first report on miR-208a, it was discovered that the βMHC gene also contains an intronic miR-208 isoform, named miR-208b (van Rooij *et al*, 2009). Interestingly, myosin and subsequent miR-208 (myomiR) expression differs significantly between species. While αMHC/miR-208a is the predominant cardiac myosin in rodents, expression of βMHC/miR-208b is more prevalent in larger mammals. Hence, additional studies in non-human primates will be important in pinpointing whether pharmacological inhibition of miR-208b in larger mammals induces comparable gene changes and biological effects as miR-208a inhibition does in rodents.

## Therapeutic inhibition of miR-122 for the treatment of HCV infection

miR-122 is a highly abundant, liver-expressed miRNA that is completely conserved from zebrafish to man and implicated in the regulation of hepatic cholesterol, lipid and iron metabolism and in maintaining liver cell identity (Lagos-Quintana *et al*, 2002; Krützfeldt *et al*, 2005; Wienholds *et al*, 2005; Esau *et al*, 2006; Elmén *et al*, 2008a,b; Castoldi *et al*, 2011; Jopling, 2012). AntimiR-mediated inhibition of miR-122 in mice results in derepression of predicted target mRNAs in the liver and lowering of plasma cholesterol by 30–40%, suggesting that miR-122 could be a potential target for cholesterol lowering (reviewed in: Rottiers & Näär, 2012). Krützfeldt *et al* (2005) were the first to report on miR-122 antagonism in mice using intravenously injected antagomirs (three doses of 80 mg/kg antagomir-122), whereas Esau *et al* (2006) used an intraperitoneally delivered, 2′ MOE-modified antimiR for inhibition of miR-122 in high-fat-diet-fed mice by treating the animals for 4 weeks with 2 weekly doses ranging from 12.5 to 75 mg/kg/dose. In a third miR-122 inhibition study, treatment of high-fat-diet-fed mice with a 15-mer LNA-modified antimiR (miravirsen) twice weekly at 5 mg/kg/dose for 6 weeks resulted in long-lasting decrease in serum cholesterol (Elmén *et al*, 2008b). Furthermore, systemic administration of this antimiR to African green monkeys at doses ranging from 1 to 10 mg/kg with three i.v. infusions over 5 days resulted in sequestration of mature miR-122 and dose-dependent and long-lasting decrease of circulating cholesterol levels, which gradually returned to baseline levels over a 3-month period after treatment (Elmén *et al*, 2008b). Importantly, short-term pharmacological inhibition of miR-122 was shown to be reversible and well tolerated in mice and non-human primates without any acute or subchronic toxicities (Elmén *et al*, 2008a,b). However, two recent studies reported that chronic loss of miR-122 function in *Mir122* germline knockout and liver-specific knockout mice, respectively, resulted in increased incidence of steatohepatitis and hepatocellular carcinoma with age (Hsu *et al*, 2012; Tsai *et al*, 2012). Hence, additional studies are required to assess the potential risks associated with long-term inhibition of miR-122. Furthermore, inhibition of miR-122 has been shown to lower both LDL cholesterol and HDL cholesterol levels mice and non-human primates (Elmén *et al*, 2008b), which implies that miR-122 is not a good therapeutic target for reducing increased levels of LDL cholesterol in hypercholesterolemia.

Apart from its role in modulating cholesterol metabolism, miR-122 was shown to function as an important host factor for hepatitis C virus (HCV) propagation by an unusual mechanism, in which two miR-122 molecules interact with the 5′ untranslated region (UTR) of the HCV genome by binding to two miR-122 seed sites in association with Ago2 (Jopling *et al*, 2005; Machlin *et al*, 2011; Shimakami *et al*, 2012a). By forming a ternary miR-122-HCV RNA complex, miR-122 protects the HCV 5′ UTR from nucleolytic degradation and thereby promotes viral RNA stability and propagation (Jopling *et al*, 2005; Machlin *et al*, 2011; Shimakami *et al*, 2012b; Mortimer & Doudna, 2013). Interestingly, inhibition of miR-122 function in cultured liver cells results in marked suppression of HCV RNA accumulation, implying that miR-122 could be a potential target for treatment of HCV infection (Jopling *et al*, 2005). Furthermore, both miR-122 binding sites are conserved in all six HCV genotypes (Li *et al*, 2011; Shimakami *et al*, 2012b), which implies that an antimiR-based HCV therapy would be genotype independent. Indeed, potent antiviral activity against all six HCV genotypes was recently reported in cultured cells using the LNA-modified antimiR miravirsen (Li *et al*, 2011), which would support its potential use for the treatment of all HCV genotype infections.

The therapeutic potential of miravirsen as a new antiviral treatment strategy was initially assessed in an efficacy study in chimpanzees, in which four animals with a chronic HCV genotype 1 infection were treated with 12 weekly i.v. doses of miravirsen with two chimpanzees receiving 5 mg/kg/dose and two receiving 1 mg/kg/dose, respectively (Lanford *et al*, 2010). A marked and long-lasting decline of viral titer was detected in the high dose treatment group with a maximum reduction of 2.6 orders of magnitude in HCV RNA levels 2 weeks after last dose with no evidence of viral rebound during the miravirsen treatment phase or side effects in the treated animals (Lanford *et al*, 2010). Furthermore, no escape mutations were detected in the two miR-122 binding sites of the HCV 5′ UTR, implying that miravirsen has a high barrier to HCV resistance (Lanford *et al*, 2010).

Two companies are currently developing antimiR-122-based therapeutics for the treatment of HCV infection. The *N*-Acetylgalactosamine (GalNAC)-conjugated antimiR-122 compound RG-101 developed by Regulus Therapeutics recently commenced dosing in healthy volunteers in a phase 1 study (http://www.regulusrx.com), whereas miravirsen, the first miRNA-targeted drug that advanced to clinical trials, is being developed by Santaris Pharma (http://www.santaris.com). Data from phase 1 safety studies in healthy male volunteers showed that miravirsen treatment was safe and well tolerated, consistent with data obtained from rodent and non-human primate studies (Elmén *et al*, 2008b; Hildebrandt-Eriksen *et al*, 2009, 2012; Lanford *et al*, 2010). Furthermore, data from the first phase 2 study with miravirsen in HCV-infected patients were recently published (Janssen *et al*, 2013). A total of 36 treatment-naïve patients with chronic HCV genotype 1 infection were enrolled for this study and randomly assigned to receive miravirsen at doses of 3, 5 or 7 mg/kg or placebo as a total of 5 weekly subcutaneous injections over 29 days. Treatment with miravirsen resulted in a dose-dependent and long-lasting antiviral activity with a mean–maximum decrease in HCV RNA levels (log10 IU/ml) of 3.0 for patients receiving 7 mg/kg and 2.9 for those receiving 5 mg/kg, compared with a decline of 0.4 observed in the placebo group. No viral resistance-associated mutations were detected in the miR-122 seed sites of HCV 5′ UTR in any of the patients. Interestingly, during the 14-week follow-up period, one patient in the 5 mg/kg group and four patients in the 7 mg/kg group had undetectable HCV RNA levels. However, four of these patients showed a viral rebound by the end of the study, implying that a 4-week miravirsen monotherapy is not sufficient to achieve a sustained virologic response (Janssen *et al*, 2013). Miravirsen was well tolerated, and there were no dose-limiting toxic effects or treatment discontinuations due to adverse events. The reported adverse events were infrequent and mostly mild, such as headache, coryza, fatigue and nausea, and no serious adverse events or clinically significant changes in safety tests, vital signs or electrocardiograms were observed. Two patients in the 7 mg/kg miravirsen treatment group showed injection-site reactions that included a combination of local erythema, itching and persistent induration. However, these were self-limited or resolved with minimal treatment, and no systemic allergic reactions were observed. (Janssen *et al*, 2013). In summary, these data show that short-term use of miravirsen in patients with chronic HCV genotype 1 infection is safe and well tolerated and provides long-lasting antiviral activity without evidence of viral resistance. Furthermore, this study implies that pharmacological modulation of miRNA activity is indeed a feasible therapeutic strategy in human patients and thus represents a landmark achievement in the development of miRNA therapeutics.

**Pending issues**

Development of improved *in vitro* and *in vivo* models of human disease and technologies for miRNA target validation.

Identification and characterization of direct miRNA targets and the impact of miRNA modulation on the target mRNAs.

Improved understanding of the antimiR oligonucleotide mechanism of action.

Development of improved delivery technologies for miRNA mimics and antimiR oligonucleotides.

Understanding of the long-term effects of miRNA modulation *in vivo*.

Comprehensive analyses of the off-target effects of miRNA-based drugs.

Pharmacokinetic/pharmacodynamics modeling of miRNA-based drugs.

Assessment of the efficacy and safety of miRNA-based therapeutics in human subjects.

## Concluding remarks

Since the seminal discovery of the first miRNA, lin-4, in the nematode *C. elegans* two decades ago, over 30,000 miRNAs have been identified in 206 species, including 2,578 mature miRNAs in humans. Indeed, miRNAs are involved in the regulation of most, if not all biological processes in the cell. Furthermore, miRNAs are frequently deregulated in human diseases, indicating that they could serve as viable targets for therapeutics. Two main strategies are employed for pharmacological modulation of miRNA activity *in vivo*: (i) restoring the function of a miRNA using either synthetic miRNA mimics or viral expression constructs, and (ii) inhibition of miRNA function by chemically modified antimiR oligonucleotides. Apart from delivery to the kidney and liver, many additional peripheral tissues have been successfully targeted using currently available delivery approaches for miRNA replacement therapies and antimiR oligonucleotides. However, the ubiquitous expression patterns reported for many miRNAs increases the risk of off-target effects by a miRNA modulator especially in indications that require chronic treatment. Thus, delivery of miRNA modulators to the cell type or tissue of interest is a key factor for successful development of miRNA-based therapeutics. One possible approach would be a conjugation strategy with the nucleic acid linked to targeting molecules, such as peptides, antibodies or other bioactive molecules, which may promote homing of the miRNA modulator to specific cell types. Alternatively, the antimiR or miRNA mimic could be encapsulated into a lipid-based formulation that enhances cell-specific uptake. Until the methods for more specific delivery become a reality, device-based delivery approaches, such as stents or catheters, local injections or ectopical delivery could be applied to circumvent some of the delivery issues. To date, delivery of antisense oligonucleotides has been reported in the lung by inhalation, to the gut by enema formulation, to the brain by intraventricular or intrathecal administration, and to the eye by direct intraocular delivery. Apart from efficient, targeted delivery, understanding the side effects and potential off-target effects alongside physiological repercussions of long-term miRNA modulation *in vivo* is of key importance. Furthermore, due to the variation in miRNA expression levels across different cell and tissue types under normal physiological conditions as well as in disease, extensive preclinical studies are required to determine the optimal level of inhibition for a given miRNA target. Similarly, development of miRNA replacement therapies will require optimization for restoring the activity of a down-regulated or lost miRNA, while preventing the introduction of supraphysiological levels of the same miRNA. Nevertheless, as described in this review, pharmacological modulation of disease-associated miRNAs has demonstrated promising therapeutic potential and appears to be well tolerated based on data from short-term studies in animal disease models and human patients. Notably, data from the first clinical phase 2 study showed that the antimiR-122 drug miravirsen was safe and well tolerated and provided prolonged antiviral activity in chronically HCV-infected patients, which implies that miRNA-based therapeutics can indeed become a reality in clinical medicine.

**Glossary**

**AAV**

Adeno-associated viruses (AAVs) are small, non-enveloped, single-stranded DNA viruses that belong to the parvovirus family and require a helper virus, such as adenovirus, herpes simplex virus or vaccinia virus to replicate

**Antagomir**

Antagomirs are 3′ cholesterol-conjugated, 2′-*O*-methyl-modified antisense oligonucleotides that inhibit miRNA function. Antagomirs are fully complementary to mature miRNAs

**antimiR**

Chemically modified, single-stranded antisense oligonucleotides that inhibit miRNA function. The length of antimiRs ranges from seed-targeting 8-mer oligonucleotides to antimiRs that are fully complementary to mature miRNAs

**Atherosclerosis**

Atherosclerosis is a condition in which an artery wall thickens as a result of the accumulation of calcium and fatty materials such as cholesterol and triglycerides

**Coryza**

Acute inflammation of the mucous membrane of the nasal cavities

**Diastolic heart failure**

Diastolic heart failure is a condition in which a decline in performance of one or both ventricles during diastole leads to symptoms of congestion from the inappropriate upward shift of the diastolic pressure–volume relation

**Erythema**

Redness of the skin or mucous membranes, caused by hyperemia of superficial capillaries

**Hypercholesterolemia**

Hypercholesterolemia is a condition characterized by very high levels of cholesterol in the blood

**Hyperemia**

Increase of blood flow to different tissues in the body

**Locked nucleic acid (LNA)**

A high-affinity RNA analogue, in which the ribose sugar is locked in a C3′-endo conformation by introduction of a 2′-O,4′-C methylene bridge

**miRISC**

The miRNA-induced silencing complex (miRISC) is a multicomponent ribonucleoprotein complex comprising a member of the Argonaute (Ago) family proteins and a mature miRNA incorporated into Ago, in addition to a number of accessory factors. The mature miRNA serves as a guide molecule for miRISC by directing it to partially complementary target sites located predominantly in the 3′ untranslated regions (UTRs) of target mRNAs in metazoans, resulting in translational repression and mRNA degradation of the targets

**miRNA mimic**

A small, chemically modified double-stranded RNA that mimics the function of an endogenous miRNA

**miRNA replacement therapy**

A therapeutic approach to treat human disease, in which the function of a lost or down-regulated miRNA is restored by introduction of a synthetic miRNA to the diseased tissue The synthetic miRNA mimic counteracts disease-dependent processes and induces a therapeutic response

**miRNA seed region**

The miRNA seed region comprises nucleotides 2-7 in the mature miRNA. An important determinant in miRNA target recognition is based on Watson–Crick base pairing of the miRNA seed region with a complementary seed match site in the target 3′ UTR

**miRNA sponge**

miRNA sponges are transcripts expressed from strong promoters, containing multiple, tandem binding sites to a miRNA of interest, thereby acting as competitive inhibitors of miRNA function

**Mirtron**

Mirtrons are short hairpin introns that provide an alternative source for animal miRNA biogenesis and use the splicing machinery to bypass Drosha cleavage in initial maturation

**Morpholino**

Morpholino oligomers are a class of chemically modified antisense oligonucleotides, in which six-membered morpholine rings replace the sugar moieties and non-ionic phosphorodiamidate linkages replace the phosphate linkages

**Peptide nucleic acid (PNA)**

PNA oligomers are uncharged oligonucleotide analogues, in which the sugar–phosphate backbone has been replaced by a peptide-like backbone consisting of N-(2-aminoethyl)-glycine units

**Steatohepatitis**

A liver disease, characterized by inflammation of the liver with concurrent fat accumulation in liver

**Toll-like receptors**

Toll-like receptors (TLRs) are a family of pattern-recognition receptors in mammals that can discriminate between chemically diverse classes of microbial products, including bacterial cell-wall components. TLRs play a key role in the innate immune system

## For more information

A comprehensive Web-accessible resource of microRNA target predictions and expression profiles: http://www.microrna.org.

miRBase—A searchable database of published miRNA sequences and annotation: http://www.mirbase.org.

TargetScan—Online software for prediction of microRNA targets: http://www.targetscan.org.

A comprehensive resource of miRNA deregulation in various human diseases: http://www.mir2disease.org.

A database of publicly and privately supported clinical studies conducted around the world, including clinical trials with miRNA-based therapeutics: http://www.clinicaltrials.gov.
